# The Effects of the Pre-Anodized Film Thickness on Growth Mechanism of Plasma Electrolytic Oxidation Coatings on the 1060 Al Substrate

**DOI:** 10.3390/ma16175922

**Published:** 2023-08-30

**Authors:** Wanting Gong, Ruina Ma, An Du, Xue Zhao, Yongzhe Fan

**Affiliations:** 1 School of Materials Science and Engineering, Hebei University of Technology, Tianjin 300130, China; gwt15665888639@163.com (W.G.); duan@hebut.edu.cn (A.D.); zhaoxue@hebut.edu.cn (X.Z.); fyz@hebut.edu.cn (Y.F.); 2 Key Laboratory for New Type of Functional Materials in Hebei Province, Tianjin 300130, China

**Keywords:** aluminum alloy, plasma electrolytic oxidation, soft sparking, pre-anodized

## Abstract

To increase the density of the micro-arc oxide coating, AA 1060 samples were pretreated with an anodic oxide film in an oxalic acid solution. Plasma electrolytic oxidation (PEO) was performed to investigate the effect of the thickness of the pre-anodic oxide film on the soft-sparking mechanism. The experimental results revealed that the PEO coating phases with different thicknesses of the pre-anodized films contained both Al and gamma–alumina (γ-Al_2_O_3_). The pre-anodized film changes the final morphology of the coating, accelerating the soft sparking transition and retaining the soft sparking. At a pre-anodized film thickness of ≤7.7 μm, the anodized films thickened before being broken through. When the pre-anodized film thickness was ≥13.1 μm, partial dissolution of the anodized films occurred before they were struck through. Two growth mechanisms for PEO coatings with different pre-anodized film thicknesses were proposed.

## 1. Introduction

Aluminum (Al) is the second most extensively utilized material globally (following steel). Nevertheless, the oxide film on the Al surface is easily broken in the corrosive medium, resulting in poor corrosion resistance and limiting the application of Al. For the sake of raising the corrosion resistance of Al, plasma electrolytic oxidation (PEO) can be adopted to add thickness to the oxide film. PEO, which is also being referred to as micro-arc oxidation (MAO), is a surface modification technique on the foundation of ordinary anodic oxidation in which metals such as Al, Mg, and Ti or their alloys are placed in an alkaline electrolyte under a high applied voltage to generate ceramic coatings on the material in situ [1,2,3,4]. The coating is strongly bonded to the substrate and can effectively enhance the wear resistance, corrosion, thermal shock, and insulation properties of the alloy surface [5,6,7]. As the applied voltage is gradually increased, the Al alloy surface rapidly generates oxide film. As the anodic voltage increases, some of the weak spots in the initial coating become breakdown sites, leading to plasma discharge, with the micro-discharge (MD) behavior significantly influencing the coating structure [8,9,10,11,12]. The high anode voltage applied during PEO results in continuous MDs throughout the reaction. This intense and complex reaction process is accompanied by rapid melting and cooling of the coatings and gas precipitation; thus, defects such as holes and cracks are often present in the prepared coatings [11,12,13]. To improve the structure of PEO coatings, strong discharge behavior should be avoided in the later stages of the reaction. Numerous studies have indicated that soft sparking (soft discharges) can occur during PEO when a highly negative current density is applied during the reaction process [14,15]. Soft sparking is accompanied by a decreasing anode voltage, a weakening of the discharge behavior, and a homogenous discharge distribution. The microstructure of the coatings is improved, and the density is increased, enhancing the corrosion resistance. In a previous study, Jaspard et al. [16] discovered the soft-sparking phenomenon and found that it only occurs when the ratio of the anode charge to the cathode charge (*R_pn_*) is <1. Additionally, with higher current density and higher AC power frequencies, the transition to soft sparking is significantly accelerated [16,17]. However, no unified theory has been established to explain the occurrence of soft sparking; hence, this phenomenon still needs to be explored [18,19,20,21,22]. Matykina et al. [23] proposed the anodization of Al in oxalic, sulfuric, or phosphoric acid solutions to produce an anodic oxide film of a certain thickness, which can then be treated with PEO to produce a coating on an Al substrate with a pre-anodic oxide film. Studies have shown that anodic oxide films do not change the final appearance of the coating. This is a very obvious discrepancy with our experimental results. To further investigate the impact of pre-anodized film thickness on the soft spark and growth mechanism of PEO coatings, an experiment was conducted on Al substrates with different thicknesses of pre-anodized films with PEO. The thickness of the pre-anodized films can be easily varied because of the low pore density of the films formed in oxalic and phosphoric acids and the wide range of thicknesses generated in oxalic acid [24,25]. Therefore, to better investigate the effect of the pre-anodized film thickness on the soft sparking state, an oxalic acid solution was selected as the anodizing solution. The effect of the pre-anodized film thickness on the soft sparking and the growth mechanism of PEO coatings were investigated by comparing the voltage–time response curves and surface/cross-sectional morphologies of the coatings at different pre-anodized film thicknesses.

## 2. Materials and Methods

### 2.1. Materials and Sample Preparation Method

The 1060 Al alloy was used as the matrix material, and its chemical composition is presented in Table 1. The material was cut into square slices with dimensions of 10 mm × 10 mm × 1.5 mm attached to a handle with dimensions of 1.5 mm × 2 mm × 35 mm. The substrate was sanded with silicon carbide (SiC) sandpaper and polished to obtain a flat surface (*R_a_* = 0.118 ± 0.005 μm), followed by washing with distilled water to eliminate residual SiC particles. To remove oil from the substrate surface, an alkaline wash treatment with a NaOH solution and a light treatment with an HF solution were applied. The specimens were rinsed with distilled water and air-dried. The dried specimens were anodized in a 40 g/L oxalic acid solution at a constant direct current voltage of 70 V. To obtain anodizing film of different thicknesses, the samples were anodized for 300, 600, 900, and 1800 s. Anodic oxide films with thicknesses of 3.3, 7.7, 13.1, and 15.5 μm were obtained, and then the substrates with anodic oxide films were subjected to PEO treatment for 900 s in the constant-current mode. The specimen without pretreatment was designated as S0, and the remaining specimens were designated as S3.3, S7.7, S13.1, and S15.5, according to the pre-anodized film thicknesses.

**Figure 1:**
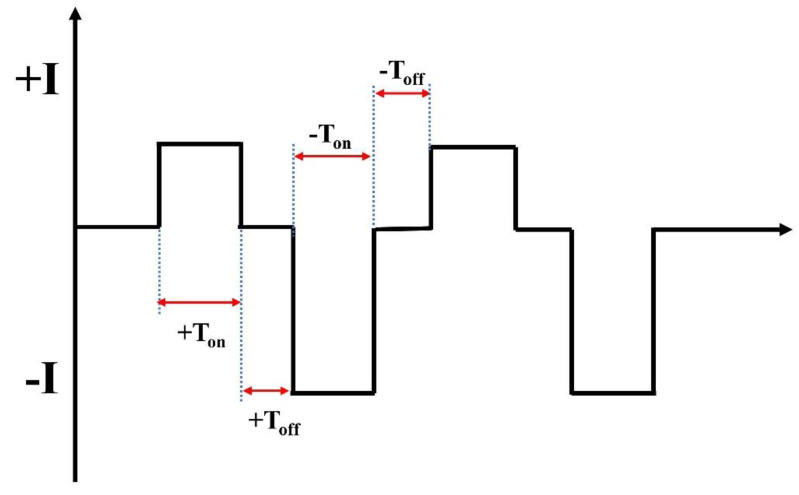
Schematic of the electrical signal waveform.

Figure 1 shows the PEO waveform, and Table 2 presents the electrolyte composition and electrical parameters. All the reagents were of analytical research grade. The electrolyte conductivity and the pH of the electrolytes were measured using a conductivity meter (HQ2200, Hach, Ames, IA, USA). The bipolar power system (SOYI-60050DM (Shanghai Suoyi Company, Shanghai, China)) used the sample and a stainless steel vessel as the working (positive) and opposite (negative) electrodes, respectively. The temperature of the electrolyte was maintained at 30 ± 3 °C using a circulated cooling system.

The PEO specimens were subjected to electrochemical testing using a Shanghai Chenhua CHI −660E electrochemical workstation (Shanghai, China). The test solution was 3.5 wt.% NaCl in water, and the test area was 1 cm^2^. A typical three-electrode measurement system was used, with the specimen as the working electrode, a saturated polymeric electrode as the reference electrode, and a Pt electrode (10 mm × 10 mm) as the auxiliary electrode. The scanning speed was 0.4 mV/s.

### 2.2. Coating Characterizations

**Figure 2:**
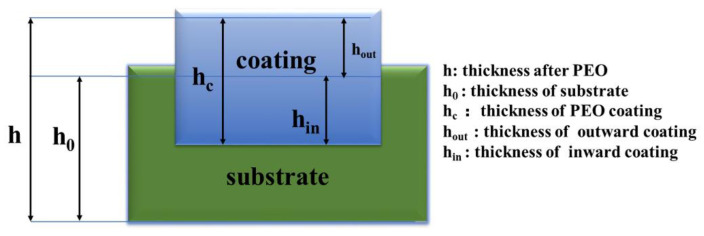
Thickness characterization schematic.

X-ray diffraction (XRD; SmartLab 9 kW (Rigaku Corporation, Tokyo, Japan)) was applied to analyze the phases of the PEO coatings. The XRD used Kα radiation with a Copper (Cu) target in steps of 0.02° and a scan range of 10° to 90° at 6 kV. The GIXRD (SmartLab 9 kW) was taken to determine the phase of the anodic oxide film. The grazing incidence angle was 0.5° and the rest of the parameters were unchanged. The thicknesses of the coatings were measured using a micrometer (model Art. Nr.64200) and an optical microscope (Olympus-BH, Shinjuku, Japan). This microscope was also used to examine the surface morphologies of the coatings and measure their roughness. Figure 2 shows a representation of the thickness parameters. The surface, cross-sectional properties and morphology of the PEO coatings were obtained using a focused ion beam (SEM; TESCAN GAIA3, Brno, The Czech Republic).

## 3. Results and Discussion

### 3.1. Voltage–Time Response

**Figure 3:**
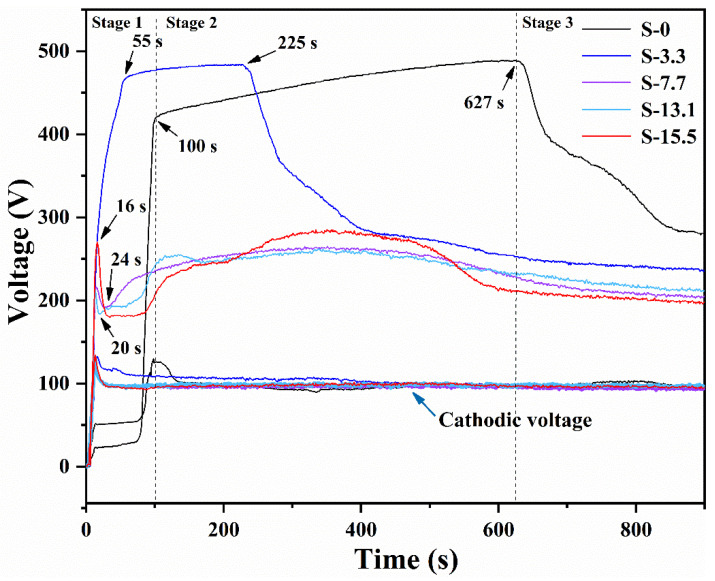
Voltage vs. time plots for the PEO process.

Figure 3 exhibits the voltage–time response curves for PEO specimens with different pre-anodized film thicknesses at a constant current, and the voltage–time response curves changed with respect to the thicknesses of the pre-anodized oxide films. The PEO process for a specimen without a pre-anodized film (specimen S0) was divided into three stages depending on the voltage change during the PEO process. Stage 1: the anodic oxide film was formed rapidly, and the voltage increased quickly. The voltage increased rapidly to 481 V within 100 s, and it reached a maximum value of 488 V at 627 s. Stage 2: dielectric breakdown occurred in the coatings, when the voltage was above the critical voltage for breakdown of the anodic oxide film. Small sparks were seen on the surface of the coating. Over time, the surface sparks on the specimen became larger. Numerous bubbles continuously escaped from the surface of the specimen, accompanied by a loud sound. Stage 3: the voltage dropped, and the PEO process progressed into a soft state.

When the pre-anodized film thickness was thin (specimen S3.3), the voltage variation of the specimen was similar to that of the specimen without the pre-anodized film. Stage 1 of specimen S3.3 was shortened by 45 s compared with that of specimen S0. At 12 s, small, uniform orange sparks appeared on the surface of specimen S3.3. Compared with specimen S0, stage 2 of specimen S3.3 was shortened by 357 s, and stage 3 of specimen S3.3 was shortened by 402 s. Compared with the voltage–time response curve for specimens S0 and S3.3, the curves for specimens S7.7, S13.1, and S15.5 exhibited different trends. The voltages of specimens S7.7, S13.1, and S15.5 increased rapidly to approximately 270 V before decreasing significantly. Subsequently, the voltage was slightly raised. In the early stages, small sparks of white were found on the surfaces of specimens S7.7, S13.1, and S15.5, and they remained until the end of the PEO process. This indicated that the soft sparking was maintained throughout the PEO process.

The reaction time between stages 1 and 2 was reduced, the voltage growth rate in stage 2 was reduced, and the sparks were appeared earlier due to the increased thickness of the pre-anodized film. As the pre-anodized film thickness increased beyond 7.7 μm, the voltage was reduced. The soft sparking was observed earlier and was maintained throughout the process. As the pre-anodized film grew thicker, the voltage of the PEO coating reached the critical value faster, and the dielectric breakdown was faster. Owing to the presence of the pre-anodized film, the sparking was brought forward, and the PEO system was formed more quickly. The coating resistance was reduced because of the entry of protons into the coating under the cathodic current, which reduced the intensity of the plasma discharges [26,27,28,29,30]. Thus, the final voltages of the PEO process were 234, 203, 212, and 198 V for the four different pre-anodized film thicknesses compared with the final voltage of 282 V for specimen S0. Insufficient anode pulses to compensate for the residual effects of the previous cycle of cathode discharge can cause negative charge migration, reducing the plasma discharge activity [26]. However, the pre-anodized film increased the oxide content of the coating; thus, the sparks were not completely extinguished until the end of the PEO process.

### 3.2. Phase Composition

**Figure 4:**
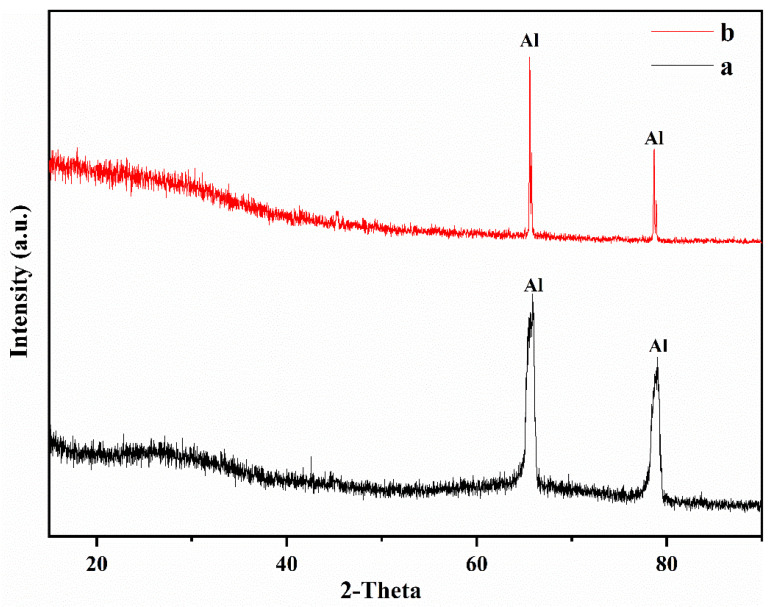
Phase composition of anodic oxide films of different thicknesses: (a) 7.7 μm, (b) 15.5 μm.

**Figure 5:**
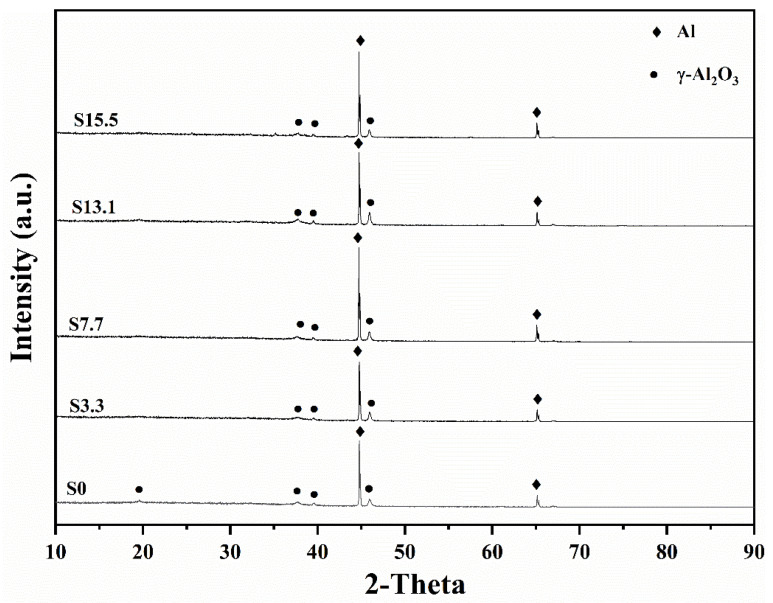
XRD patterns of PEO coatings under different thicknesses of PEO pre-anodized film.

The anodic oxide films were made of amorphous aluminum oxide and Al (Figure 4). The XRD patterns of the PEO coating surfaces with pre-anodized films of different thicknesses are given in Figure 5. The PEO coatings with different thicknesses of pre-anodized films comprised Al and γ-Al_2_O_3_, and the contents of the phase were different. The diffraction peak of Al in the XRD pattern corresponded to the Al of the substrate. Combined with the color change in the coating surface, it can be seen that the coating phase gradually changed from amorphous alumina to γ-Al_2_O_3_. The intensities of the diffraction peaks of Al for the specimens with the pre-anodized film were lower compared with the PEO coating without the pre-anodized film, owing to the thickening of the coating. In the specimens with pre-anodized films, the thickness of the PEO coating was significantly increased, along with the γ-Al_2_O_3_ content. The energy input during the reaction was reduced in the soft sparking. However, the cold quenching effect of the electrolyte was reduced and the γ-Al_2_O_3_ content was increased owing to the thicker coating, which provided a degree of “insulation”. The α-Al_2_O_3_ phase, which is related to the reaction process voltage [31,32], was not detected in the coatings. Because low voltages were used in the experiment, the intensities of the MDs were relatively low; thus, the energy requirement for the phase change was not fulfilled. The voltage at the beginning of the reaction of specimen S3.3 was high, but the resulting coating was thin at this time. Consequently, the heat transfer to the substrate was accelerated, and the temperature requirement for the γ → α-Al_2_O_3_ phase transition was difficult to be achieved.

### 3.3. Surface Microstructures of Coatings

**Figure 6:**
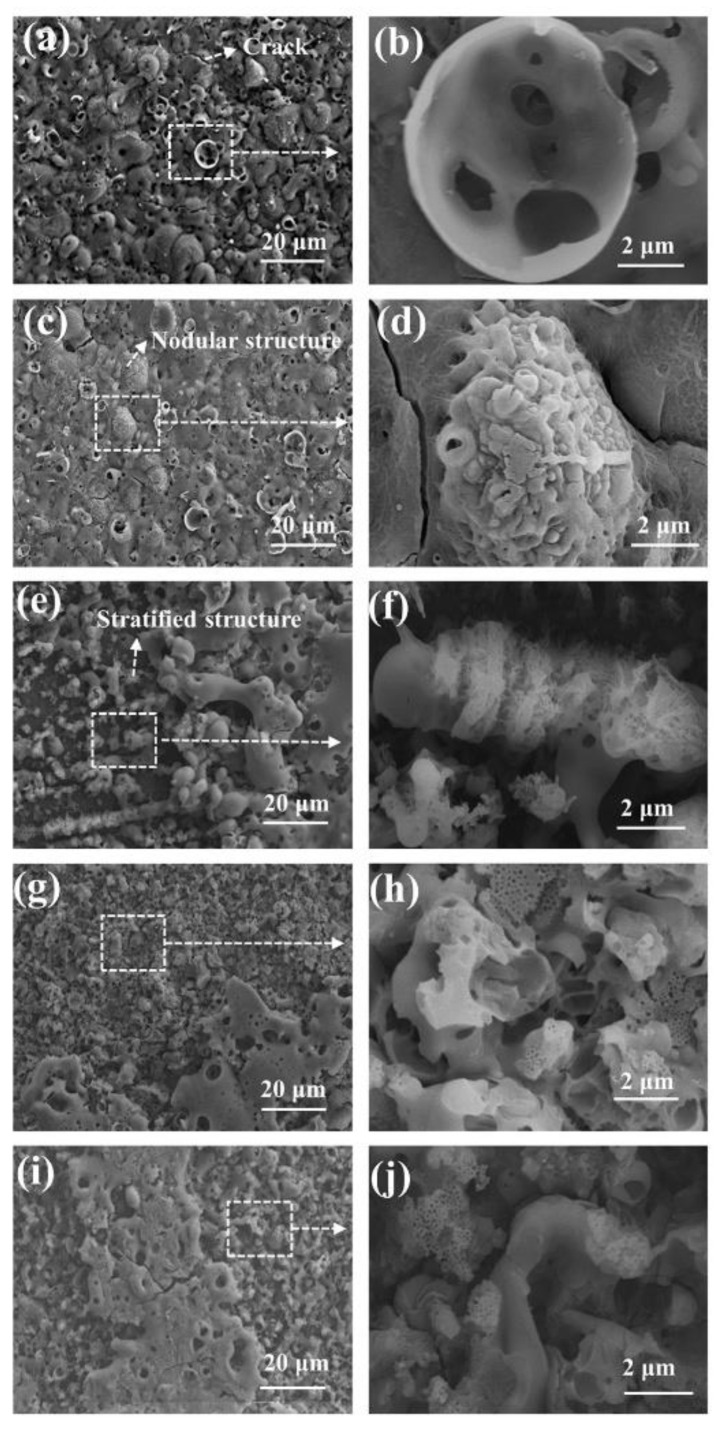
SEM images of coated surfaces: (**a**,**b**) S0; (**c**,**d**) S3.3; (**e**,**f**) S7.7; (**g**,**h**) S13.1; (**i**,**j**) 15.5.

The surface morphologies of the PEO coatings with different pre-anodized film thicknesses are exhibited in Figure 6. The coatings of specimens S0 and S3.3 exhibit pancake-like and nodular regions (Figure 6a–d). Numerous hole-like structures are present on the surfaces, and traces of multiple discharges are observed on the inside of the holes. Visible cracks exist on the coating surfaces. The nodular-like and pore-like structures on the surfaces of the PEO coatings of specimens S7.7, S13.1, and S15.5 largely disappear. Small pancake-like structures appear on the surfaces. The fragmentary pancake-like structures are surrounded by many small discharge holes. The model proposed by Hussein et al. [14] indicates that the nodular structures on the coating surface are electrolyte-rich deposited structures generated by A- and C-type discharges. The substrate is oxidized owing to a penetrating dielectric breakdown (B-discharge) at the coating/substrate interface, forming an Al-rich pancake-like structure. Cheng et al. [17] supplemented this model by proposing two models for the D-type discharge occurring in the inner and outer pores and the E-type discharge occurring at the pancake-like structure. During the PEO process, the coating melts owing to the intense discharge, and the electrolyte has a significant “cold quenching” effect. Cracks are observed on the surfaces of the specimens owing to the large temperature difference that accompanies the solidification of the molten material [33,34,35,36]. The coating thickens owing to the existence of a pre-anodized oxide film. The occurrence of cathodic discharge is facilitated by the increased thickness of the coating. Cathodic discharge inhibits the generation of successive dielectric breakdown of the anodic discharge at the same location [30]. Therefore, the number of defects produced by continuous strong discharge is reduced.

The number of discharges within the coating increases, causing the individual holes in the surface to become larger. The plasma discharge behavior that occurs inside the coating is extended to the surface, leaving larger discharge holes at the corresponding location on the surface. The soft sparking is promoted when a pre-anodized film is present. In the soft sparking, the intensity of the plasma discharge is reduced, along with the number of B-type discharges. The reduction in the number of B-type discharges is conducive to an increase in the probability of Type D discharge occurring inside the coating, resulting in larger individual discharge holes. The presence of a pre-anodized film makes sparks appear on the coating surface earlier. The yellow anodic oxide film, which is composed of amorphous aluminum oxide, melts under the action of electric sparks. A grey–white coating is produced, and it consists of the γ-Al_2_O_3_ phase. Thus, small pancake-like structures are observed on the PEO coating surfaces of specimens S7.7, S13.1, and S15.5.

### 3.4. Coating Cross Sections and Thicknesses

**Figure 7:**
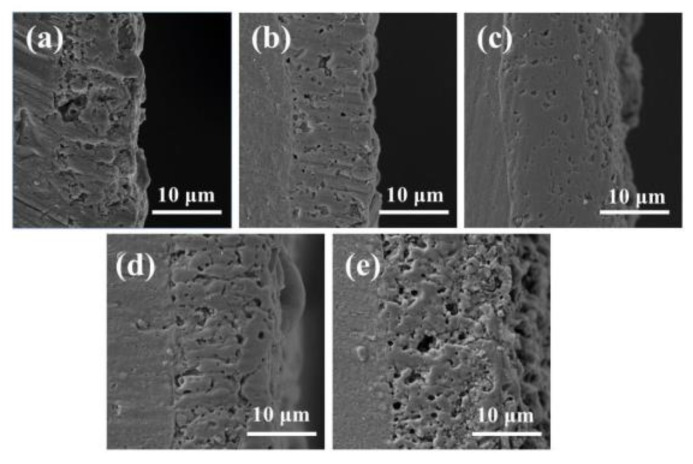
PEO cross-sectional SEM images and elemental distributions: (**a**) S0; (**b**) S3.3; (**c**) S7.7; (**d**) S13.1; (**e**) S15.5.

Figure 7 presents cross-sectional views of the coatings with pre-anodized films of different thicknesses. As depicted in Figure 7, the cross-sections have cavities and discharge channels of different sizes. The porosity of the specimen cross-section was examined by using the ImageJ1.48V software, and the results indicated that specimen S0 had the largest cavity area and S7.7 had the smallest cavity area (5.8%). In the PEO process, heat was produced by the plasma discharge. The substrate was heated to produce high-temperature molten oxide, accompanied by the generation of gases. Gases were trapped inside the coating, resulting in cavities being formed after the oxide had solidified. The number of strong discharges is reduced in soft sparking, reducing the total amount of gas produced by the plasma discharge and thus the cavity volume. The sparks were not completely extinguished during the PEO process for specimens with a certain thickness of the pre-anodized film. The continuous reaction of the soft plasma discharge increased the density of the coating. As the thickness of the pre-anodized film increased, the probability of D-type discharge increased. The D-type discharge allowed the ejection of the molten oxide. The remaining oxide in the inner layer was insufficient to fill the discharge channels, and discharge traces remained in the coating. Therefore, when the thickness of the pre-anodized film increased to a certain level, the number of coating defects was increased because the number of D-type discharges was increased.

**Figure 8:**
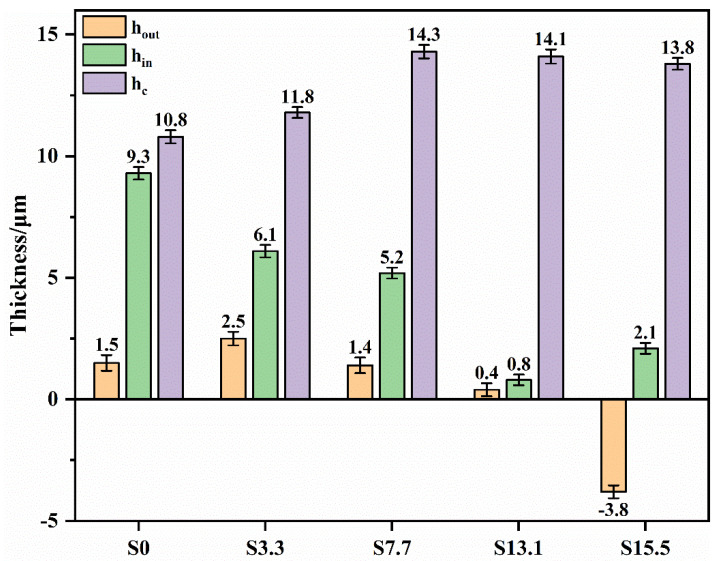
Thickness statistics of the PEO coatings.

**Figure 9:**
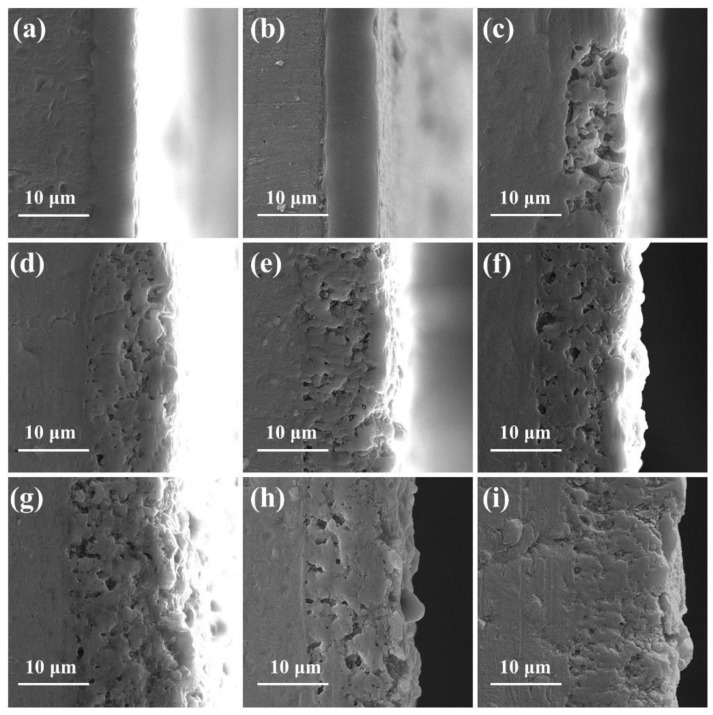
Cross-sectional SEM images of the pre-anodized film with a thickness of 7.7 μm for different PEO times: (**a**) 50 s; (**b**) 100 s; (**c**) 150 s; (**d**) 200 s; (**e**) 300 s; (**f**) 400 s; (**g**) 600 s; (**h**) 800 s; (**i**) 900 s.

**Figure 10:**
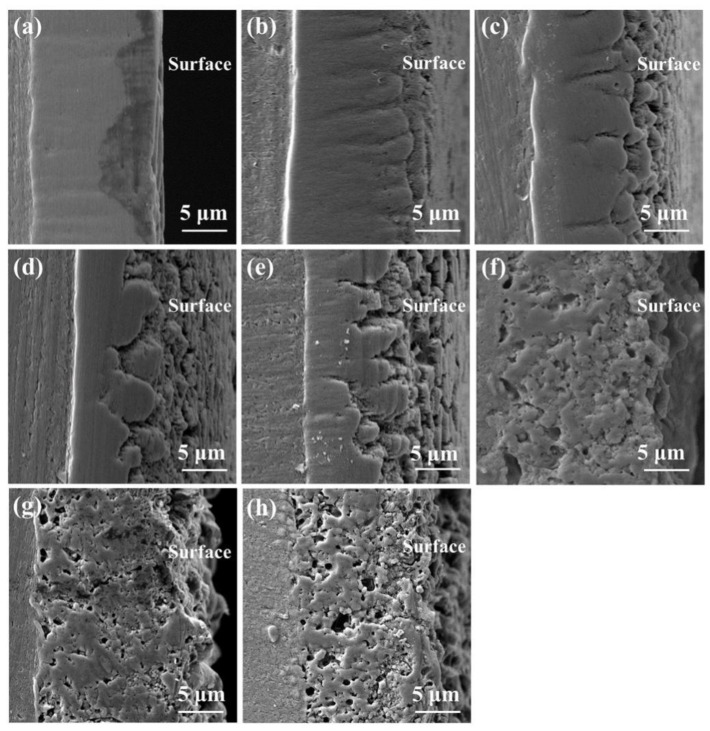
Cross-sectional SEM images of pre-anodized films with a thickness of 15.5 μm for different PEO times: (**a**) 50 s; (**b**) 100 s; (**c**) 200 s; (**d**) 300 s; (**e**) 400 s; (**f**) 600 s; (**g**) 800 s; (**h**) 900 s.

**Figure 11:**
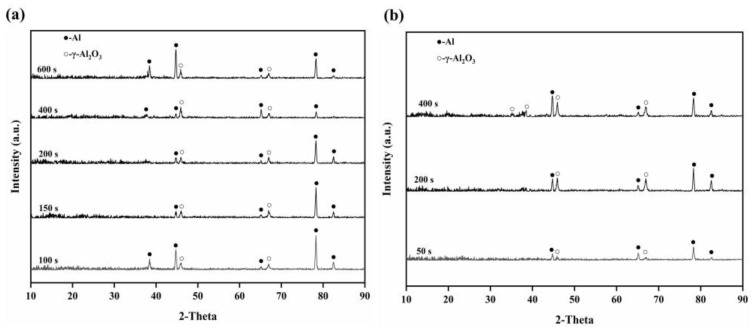
Phase composition of PEO coatings for different PEO treatment times: (**a**) specimen S7.7; (**b**) specimen S15.5.

The coating thickness variations are shown in Figure 8. Relative to specimen S0 with a thickness of 10.8 μm, the thicknesses of the PEO coatings with the pre-anodized films were larger. The maximum thickness of the coating (14.3 μm) was observed for specimen S7.7. For specimens S3.3, S7.7, and S13.1, the PEO coating was thicker than the pre-anodized film, but the rate of increase in the coating thickness exhibited a decreasing trend. The thickness of the PEO coating on specimen S15.5 was reduced by 1.7 μm compared with that of the pre-anodized film. To further investigate the impact of the pre-anodized film on the growth process of the coatings, the microstructures of the coatings of specimens S7.7 (Figure 9) and S15.5 (Figure 10) for different PEO times were analyzed. During the first 100 s, the thickness of the oxide layer of specimen S7.7 continuously increased. The presence of the pre-anodized film caused small sparks to be advanced on the surface of the specimen. The surface of the specimen was melted by the heat generated by the spark discharge. An oxide film with ceramic characteristics was formed, and the phase of the coating was transformed from amorphous aluminum oxide into γ-Al_2_O_3_ (Figure 11a). At 150 s, coating breakdown occurred when the voltage reached the critical breakdown voltage. Owing to its good insulating properties, the coating was first dielectrically broken down at the point of impurities or defects, and then the dielectric breakdown extended from the weak point of the film to the sides. The surface of the matrix was melted and oxidized. OH^−^ and other anions of electrolytes constantly entered the coating and participated in the formation of the coating through the discharge channels under the action of the electric field [36,37,38,39,40]. After 600 s, the thickness of the coating remained essentially unchanged. This is because the growth rate of the coating was limited by the cathodic discharge at this stage of the PEO process. Additionally, the coating was continuously densified under the action of the soft sparking, and the volume of the cavities and discharge channels of the coating was reduced, indicating that the densification occurred after the soft-sparking transition.

As shown in Figure 10a–e, the anodic oxide film of specimen S15.5 dissolved because of the cathodic discharge at the beginning of the PEO process. The surface roughness of the coating increased while the top anodic oxide film heated to produce a ceramic coating, and the coating surface formed a thin grayish–white transparent layer. At 50 s, γ-Al_2_O_3_ was detected in the coating (Figure 11b). When the anodic oxide film was dissolved to achieve a certain thickness, dielectric breakdown occurred in the coating. The coating was broken very evenly, so that the MDs was evenly distributed on the coating. After 800 s, the coating thickness remained essentially unchanged, but the total cavity volume increased. Thickening of the pre-anodized film resulted in enhanced cathodic polarization. Cathodic polarization lowered the potential barrier, and the conductivity of the coating continued to increase in the following anode half-cycle. The reduction in the resistivity of the PEO coating reduced the number of strong discharges and slowed the growth of the coating, increasing the number of low-energy D-type discharges. The energy of the melt generated by the D-discharges was low. The melt cooled rapidly on the coating, causing the outer pores of the coating to become filled.

### 3.5. Corrosion Behavior

**Figure 12:**
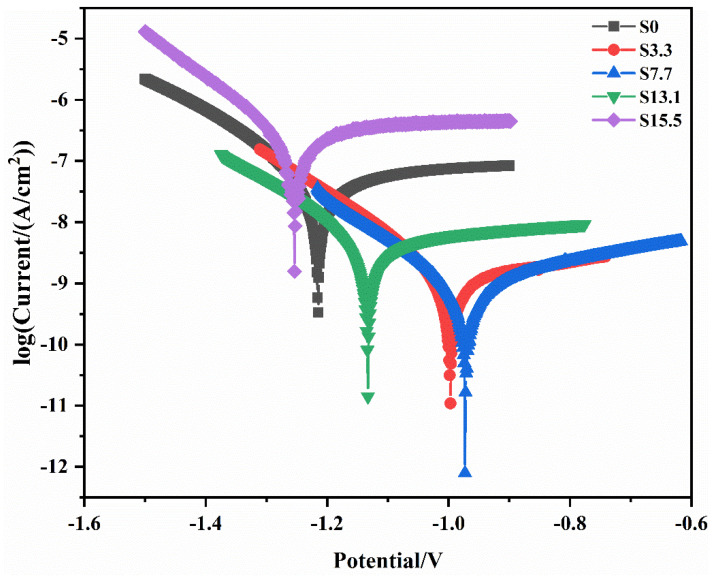
Potentiodynamic polarization curves for specimens S0, S3.3, S7.7, S13.1, and S15.5.

The Tafel curves for PEO coatings without a pre-anodized film and with pre-anodized films of different thicknesses are illustrated in Figure 12. Corrosion current density (*i_corr_*), corrosion potential (*E_corr_*), and polarization resistance (*R_ρ_*) are the main parameters for evaluating the corrosion resistance of a material. These parameters are presented in Table 3. The *R_ρ_* can be determined by using the Stern–Geary equation [32]:

(1) $$R_{\rho }=\frac{b_{c}\times b_{a}}{2.303i_{corr}(b_{c}+b_{a})}$$

Usually, the corrosion resistance and *i_corr_* show a negative correlation. Compared with specimen S0, the *i_corr_* was reduced by at least one order of magnitude at a pre-anodized film thickness of ≤13.1 μm. Additionally, the *E_corr_* was increased, and the corrosion resistance was improved. Among the specimens, S7.7 had the lowest current density; thus, it had the best corrosion resistance.

The *i_corr_* of specimen S7.7 was two orders of magnitude lower than that of specimen S0. When the thickness of the pre-anodized film increased beyond 13.1 μm, the thickness of the pre-anodized film increased, increasing the *i_corr_* of the coating and reducing the *R_ρ_*. The corrosion resistance was significantly affected by the thickness and porosity of the coating. Compared with specimen S0, specimens S3.3 and S7.7 exhibited improved corrosion resistance owing to the thickening of the oxide layer and the reduced number of coating defects. When the pre-anodizing film thickened to a certain extent, the thickness of the coating increased. The reduced coating thickness and increased porosity for specimens S13.1 and S15.5 resulted in a reduction in their corrosion resistance.

### 3.6. PEO Coating Growth Mechanism under Soft Sparking

**Figure 13:**
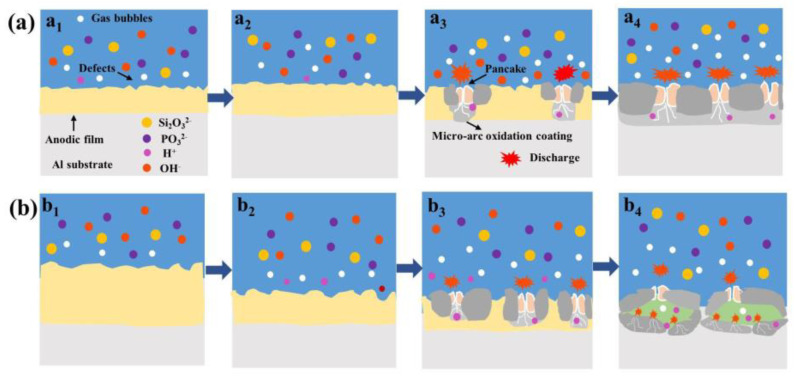
Growth model of PEO coatings under the soft-sparking mechanism with pre-anodized oxide films: (**a**) the thickness of the pre-anodized film is ≤7.7 µm; (**b**) the thickness of the pre-anodized film is ≥13.1 µm.

When the thickness of the pre-anodized film is ≤7.7 μm, the growth mechanism of the PEO coating is shown in Figure 13a. Under the action of the electric field, the anodic oxide film is thickened. Anodic polarization and cathodic polarization are induced by the application of the alternating-current pulsed power. The anions are collected on the surface of the specimen under the effect of anodic polarization, and the charging gas is used as an ignition source. Then, a spark discharge is generated on the coating surface. The topmost coating is heated and melted by sparks, forming an oxide film with ceramic characteristics. Dielectric breakdown takes place first at the defect when the voltage reaches the breakdown voltage. The dielectric breakdown gradually expands from the defect to produce PEO coatings on both sides simultaneously. Powerful MDs are linked to the cavity via cracks. MDs generates a secondary discharge at the cavities which generates gas and increases the cavity volume. The anions enter the coating and participate in the reaction through cracks, cavities, or other defects in the presence of electric fields and discharge behavior. With a pre-anodized film, the cathodic polarization is enhanced.

Cathodic polarization involves the absorption of cations into the coating, reducing its resistivity, which reduces the MD strength. High cathodic currents inhibit the occurrence of successive dielectric breakdown at the same locations; thus, the MDs are more evenly distributed as the film thickness grows. A lower resistivity of the coating facilitates the passage of the cathodic current, which promotes the HER (hydrogen evolution reaction) on the coating/solution side. This increases the alkalinity of the local solution. The increased alkalinity of the solution contributes to the formation of soluble aluminum hydroxyl complexes, which are converted into aluminum oxide in the presence of H_2_ and deposited on the coating, adding to its thickness. The pre-anodized film also provides oxides for MDs, which can prevent the sparks from being extinguished.

The reaction mechanism in the PEO process for a pre-anodized film thickness of ≥13.1 μm is shown in Figure 13b. At the beginning, the cathodic discharge is enhanced owing to the increased thickness of the film. The anodic oxide film begins to be dissolved from the surface under the action of the cathodic discharge. When the film layer is dissolved to a certain thickness, dielectric breakdown occurs in the film layer. As heat is generated through the plasma reaction, the oxide layer is heated and undergoes a phase transition, producing γ-Al_2_O_3_. The anodic oxide film is extremely thick and prevents the movement of protons in the coating, which prevents the HER at the cathode. This increases the acidity of the local solution. During anodizing polarization, acidification leads to a lower barrier and depolarization of the coating. Consequently, the MD intensity is reduced in the anode phase, and the growth of the coating slows. The reduction in the number of intense discharges increases the probability of D-type discharges, which occur in the cavities created by B-type discharges. Because of the low energy, the melt is cooled at the cavity, and the pores in the outer layer are filled. This makes it difficult for the generated gases to escape, reducing the coating density.

## 4. Conclusions

The effects of the pre-anodized film thickness on the growth mechanism of PEO coatings under soft sparking were investigated by pre-anodizing a 1060 Al substrate. Based on the experimental results, the following conclusions are obtained:

1. The pre-anodized film promoted the early occurrence of soft sparking and maintained the sparks throughout the PEO process. In the soft-sparking state, reactions constantly occurred in the coating, leading to an increase in its density.
2. The coating with the pre-anodized film consisted of Al and γ-Al_2_O_3_ phases. Early in the anodizing stage, sparks appeared on the surface of the film, and the surface layer was heated to produce γ-Al_2_O_3_. When the voltage reached the breakdown voltage, the remainder of the coating was broken through, and the phase transition of the coating occurred.
3. The presence of the pre-anodized film changes the final morphology of the coating. The pre-anodized film allowed the volume of the PEO coating cavity to be reduced, increasing the density of the PEO coating. When the thickness of the pre-anodized layer was ≥13.1 μm, the number of plasma discharges occurring inside the coating increased, and the escape of the gases generated by the reaction became difficult, increasing the volume of the cavity between the exterior and interior of the coating.
4. As the thickness of the pre-anodized film increased, the thickness of the PEO coating first increased and then decreased. At a pre-anodized film thickness of 7.7 μm, the PEO coating exhibited its maximum thickness of 14.3 μm. When the thickness of the pre-anodizing film increased beyond 13.1 μm, the PEO coating grew slowly, and its thickness decreased.

## Figures and Tables

**Table 1:** Chemical composition of the 1060 Al alloy (wt.%).

Element	Al	Si	Cu	Mg	Zn	Mn	Ti	V	Fe
Content	≥99.6	≤0.25	≤0.05	≤0.03	≤0.05	≤0.03	≤0.03	≤0.05	≤0.35

**Table 2:** Electrolyte composition and electrical parameters.

Electrolyte Information	Electrical Parameters		
Na_2_SiO_3_	3 g/L	Frequency	500 Hz
(NaPO_3_)_6_	5 g/L	Duty cycle	30%
KOH	1 g/L	Anodic current density	300 mA·cm^−2^
Conductivity	13.96 mS·cm^−1^	Cathodic current density	600 mA·cm^−2^
pH	12.1	T_on_	600 μs
		T_off_	400 μs

**Table 3:** Parameters derived from polarization curves of S0, S3.3, S7.7, S13.1 and S15.5.

Sample	*E_corr_* (V/SCE)	*i_corr_* (A/cm^2^)	*β_a_* (mV·dec^−1^)	–*β_c_* (mV·dec^−1^)	*R_ρ_* (kΩ·cm^2^)
S0	−1.2163	3.537 × 10^−8^	8.342	2.944	2.671 × 10^4^
S3.3	−1.0037	1.270 × 10^−9^	8.410	2.209	3.419 × 10^5^
S7.7	−0.9744	8260 × 10^−10^	7.459	3.785	5.260 × 10^5^
S13.1	−1.1334	4.180 × 10^−9^	7.717	2.594	1.039 × 10^5^
S15.5	−1.2560	2.066 × 10^−7^	8.189	2.634	2.102 × 10^3^

## Data Availability

The raw/processed data required to reproduce these findings cannot be shared at this time as they are part of an ongoing study.
